# Tetra­aqua­tetra­kis­(4,4′-bipyridine dioxide-κ*O*)terbium(III) octa­cyanidotungstate(V)

**DOI:** 10.1107/S1600536812005004

**Published:** 2012-02-10

**Authors:** Yu-Sheng Shi, Mao-Qian Ran, Ying-Ying Chen, Ai-Hua Yuan

**Affiliations:** a School of Biology and Chemical Engineering, Jiangsu University of Science and Technology, Zhenjiang 212003, People’s Republic of China

## Abstract

In the title compound, [Tb(C_10_H_8_N_2_O_2_)_4_(H_2_O)_4_][W(CN)_8_], both metal atoms are eight-coordinated. The Tb^III^ ion displays a dodeca­hedral geometry, while the W^v^ ion exhibits a distorted square-anti­prismatic geometry. The Tb atoms are located on a special position of site symmetry -4, whereas the W atoms are located on a twofold rotation axis. The cations are linked by O—H⋯O hydrogen bonds. The title compound is isotypic with the corresponding and previously described Mo compound [Qian & Yuan (2011 ▶). *Acta Cryst.* E**67**, m845].

## Related literature

For general background to octa­cyano­metallate-based compounds, see: Sieklucka *et al.* (2011 ▶); Zhou *et al.* (2010 ▶). For related structures, see: Qian & Yuan (2011 ▶). For the preparation of the title compound, see: Bok *et al.* (1975 ▶).
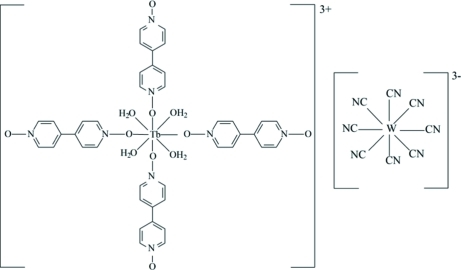


## Experimental

### 

#### Crystal data

[Tb(C_10_H_8_N_2_O_2_)_4_(H_2_O)_4_][W(CN)_8_]
*M* *_r_* = 1375.73
Tetragonal, 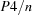

*a* = 17.9222 (7) Å
*c* = 7.8915 (6) Å
*V* = 2534.8 (2) Å^3^
*Z* = 2
Mo *K*α radiation
μ = 3.73 mm^−1^
*T* = 291 K
0.26 × 0.23 × 0.20 mm

#### Data collection

Bruker SMART APEX CCD diffractometer
Absorption correction: multi-scan (*SADABS*; Bruker, 2004 ▶) *T*_min_ = 0.444, *T*_max_ = 0.522
19073 measured reflections
2498 independent reflections
2299 reflections with *I* > 2σ(*I*)
*R*_int_ = 0.032

#### Refinement

*R*[*F* ^2^ > 2σ(*F* ^2^)] = 0.014
*wR*(*F* ^2^) = 0.037
*S* = 1.07
2498 reflections
177 parameters
H-atom parameters constrained
Δρ_max_ = 0.30 e Å^−3^
Δρ_min_ = −0.64 e Å^−3^

### 

Data collection: *SMART* (Bruker, 2004 ▶); cell refinement: *SAINT* (Bruker, 2004 ▶); data reduction: *SAINT*; program(s) used to solve structure: *SHELXS97* (Sheldrick, 2008 ▶); program(s) used to refine structure: *SHELXL97* (Sheldrick, 2008 ▶); molecular graphics: *DIAMOND* (Brandenburg, 2006 ▶); software used to prepare material for publication: *SHELXTL* (Sheldrick, 2008 ▶).

## Supplementary Material

Crystal structure: contains datablock(s) I, global. DOI: 10.1107/S1600536812005004/bx2386sup1.cif

Structure factors: contains datablock(s) I. DOI: 10.1107/S1600536812005004/bx2386Isup2.hkl

Additional supplementary materials:  crystallographic information; 3D view; checkCIF report

## Figures and Tables

**Table 1:** Hydrogen-bond geometry (Å, °)

*D*—H⋯*A*	*D*—H	H⋯*A*	*D*⋯*A*	*D*—H⋯*A*
O1—H1*B*⋯O3^i^	0.87	1.81	2.6695 (18)	167
O1—H1*A*⋯O3^ii^	0.88	1.89	2.7415 (18)	165

Symmetry codes: (i) 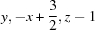; (ii) 
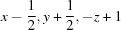
.

**Fig. 1.**
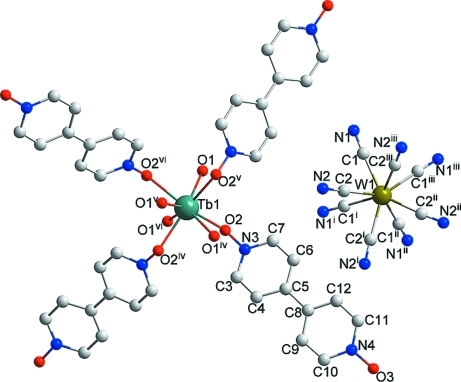
ORTEP diagram of the title compound. Hydrogen atoms have been omitted for clarity and thermal ellipsoids are presented at the 30% probability level. Symmetry codes: (i) -x + 3/2, -y + 3/2, z; (ii) -y + 3/2, x, z; (iii) y, -x + 3/2, z; (iv) y - 1/2, -x + 1, -z + 1; (v) -y + 1, x + 1/2, -z + 1; (vi) -x + 1/2, -y + 3/2, z.

## supplementary crystallographic information

### Comment

In the past few years, a considerable effort on crystal engineering has been devoted to the design and construction of octacyanometallates [*M*(CN)_8_]^3-/4-^ (*M* = Mo, W)-based magnets (Sieklucka *et al.*, 2011). The combination of [*M*(CN)_8_]^3-^ as a carrier of unpaired spin with transition- or lanthanide-metal cations has produced various dimensional molecular structures, and further displayed intriguing magnetic properties (Zhou *et al.*, 2010). Recently, we have used [W(CN)_8_]^3-^ as the building block to react with Tb^3+^ and 4,4'-bipyridine dioxide (4,4'-dpdo), obtaining a new ionic compound, [Tb(4,4'-dpdo)_4_(H_2_O)_4_][W(CN)_8_], which is isomorphous to [Tb(4,4'-dpdo)_4_(H_2_O)_4_][Mo(CN)_8_] reported previously by our group (Qian & Yuan, 2011).

In the structure, each Tb atom is located on a special position of site symmetry 4, whereas each Mo atom is located on a twofold rotation axis. The W^v^ exhibits a distorted square antiprism, while the Tb^III^ center displays an eight-coordinated decahedron geometry with 2.386 Å of the mean Tb—O bond length. The average W—C and C—N distances are 2.174 and 1.158 Å, respectively, while the W—CN bond angles are nearly linear with a maximum deviation from linearity of 4.0°. The neighboring cations are linked through O—H···O hydrogen bonds, Table 1.

### Experimental

Single crystals of the title compound were prepared at room temperature in the dark by slow diffusion of a H_2_O solution (3 ml) containing Tb(NO_3_)_3_.6H_2_O (0.05 mmol) and 4,4'-dpdo (0.05 mmol) into a CH_3_CN solution (15 ml) of [HN(*n*-C_4_H_9_)_3_]_3_[W(CN)_8_].4H_2_O (0.05 mmol) (Bok *et al.*, 1975). After two weeks, yellow block crystals were obtained.

### Refinement

All non-hydrogen atoms were refined with anisotropic thermal parameters. The H atoms of 4,4'-bpdo ligands were calculated at idealized positions with C—H = 0.93 Å and included in the refinement in a riding mode with *U*(H) set to 1.2 *U*_eq_(C). The H atoms bound to oxygen atom from coordinated water molecule were located from difference maps and refined as riding with O—H = 0.85 Å and *U*(H) set to 1.2*U*_eq_(O).

### Crystal data

**Table:**

[Tb(C_10_H_8_N_2_O_2_)_4_(H_2_O)_4_][W(CN)_8_]	*D*_x_ = 1.802 Mg m^−^^3^
*M**_r_* = 1375.73	Mo *K*α radiation, λ = 0.71073 Å
Tetragonal, *P*4/*n*	Cell parameters from 9955 reflections
Hall symbol: -P 4a	θ = 2.3–27.5°
*a* = 17.9222 (7) Å	µ = 3.73 mm^−^^1^
*c* = 7.8915 (6) Å	*T* = 291 K
*V* = 2534.8 (2) Å^3^	Block, yellow
*Z* = 2	0.26 × 0.23 × 0.20 mm
*F*(000) = 1350	

### Data collection

**Table:**

Bruker SMART APEX CCD diffractometer	2498 independent reflections
Radiation source: fine-focus sealed tube	2299 reflections with *I* > 2σ(*I*)
Graphite monochromator	*R*_int_ = 0.032
φ and ω scans	θ_max_ = 26.0°, θ_min_ = 1.6°
Absorption correction: multi-scan (*SADABS*; Bruker, 2004)	*h* = −22→22
*T*_min_ = 0.444, *T*_max_ = 0.522	*k* = −22→22
19073 measured reflections	*l* = −9→9

### Refinement

**Table:**

Refinement on *F*^2^	Primary atom site location: structure-invariant direct methods
Least-squares matrix: full	Secondary atom site location: difference Fourier map
*R*[*F*^2^ > 2σ(*F*^2^)] = 0.014	Hydrogen site location: inferred from neighbouring sites
*wR*(*F*^2^) = 0.037	H-atom parameters constrained
*S* = 1.07	*w* = 1/[σ^2^(*F*_o_^2^) + (0.0164*P*)^2^ + 1.703*P*] where *P* = (*F*_o_^2^ + 2*F*_c_^2^)/3
2498 reflections	(Δ/σ)_max_ = 0.001
177 parameters	Δρ_max_ = 0.30 e Å^−^^3^
0 restraints	Δρ_min_ = −0.64 e Å^−^^3^

### Special details

**Table:**

Geometry. All e.s.d.'s (except the e.s.d. in the dihedral angle between two l.s. planes) are estimated using the full covariance matrix. The cell e.s.d.'s are taken into account individually in the estimation of e.s.d.'s in distances, angles and torsion angles; correlations between e.s.d.'s in cell parameters are only used when they are defined by crystal symmetry. An approximate (isotropic) treatment of cell e.s.d.'s is used for estimating e.s.d.'s involving l.s. planes.
Refinement. Refinement of *F*^2^ against ALL reflections. The weighted *R*-factor *wR* and goodness of fit *S* are based on *F*^2^, conventional *R*-factors *R* are based on *F*, with *F* set to zero for negative *F*^2^. The threshold expression of *F*^2^ > σ(*F*^2^) is used only for calculating *R*-factors(gt) *etc*. and is not relevant to the choice of reflections for refinement. *R*-factors based on *F*^2^ are statistically about twice as large as those based on *F*, and *R*- factors based on ALL data will be even larger.

### Fractional atomic coordinates and isotropic or equivalent isotropic displacement parameters (Å^2^)

**Table:**

	*x*	*y*	*z*	*U*_iso_*/*U*_eq_	
W1	0.7500	0.7500	0.24882 (2)	0.01913 (5)	
Tb1	0.2500	0.7500	0.5000	0.01051 (5)	
O1	0.29389 (7)	0.81662 (7)	0.25409 (15)	0.0171 (3)	
H1B	0.3287	0.8015	0.1848	0.021*	
H1A	0.2637	0.8394	0.1839	0.021*	
N1	0.68065 (11)	0.89541 (11)	0.0386 (3)	0.0393 (5)	
C1	0.70418 (11)	0.84317 (11)	0.1061 (3)	0.0284 (5)	
O2	0.35045 (7)	0.67744 (8)	0.39890 (16)	0.0239 (3)	
N2	0.59625 (11)	0.79552 (11)	0.4621 (3)	0.0374 (5)	
C2	0.65117 (11)	0.78032 (11)	0.3929 (3)	0.0278 (4)	
O3	0.72018 (7)	0.38740 (8)	1.00441 (16)	0.0212 (3)	
N3	0.39907 (9)	0.63844 (9)	0.49170 (19)	0.0194 (3)	
C7	0.46983 (11)	0.66305 (11)	0.5066 (3)	0.0248 (4)	
H7	0.4843	0.7066	0.4515	0.030*	
C6	0.52093 (11)	0.62425 (11)	0.6029 (3)	0.0234 (4)	
H6	0.5695	0.6420	0.6125	0.028*	
C5	0.50060 (10)	0.55836 (10)	0.6866 (2)	0.0171 (4)	
C4	0.42720 (10)	0.53429 (11)	0.6643 (2)	0.0200 (4)	
H4	0.4114	0.4903	0.7157	0.024*	
C3	0.37781 (11)	0.57476 (11)	0.5672 (2)	0.0226 (4)	
H3	0.3291	0.5578	0.5538	0.027*	
C8	0.55622 (10)	0.51442 (10)	0.7837 (2)	0.0158 (4)	
C12	0.62818 (10)	0.54238 (10)	0.8120 (2)	0.0194 (4)	
H12	0.6399	0.5906	0.7774	0.023*	
C11	0.68155 (10)	0.49949 (10)	0.8903 (2)	0.0202 (4)	
H11	0.7290	0.5189	0.9089	0.024*	
N4	0.66553 (8)	0.42914 (8)	0.94064 (19)	0.0163 (3)	
C10	0.59602 (10)	0.40097 (10)	0.9223 (2)	0.0178 (4)	
H10	0.5854	0.3532	0.9613	0.021*	
C9	0.54087 (10)	0.44284 (10)	0.8461 (2)	0.0171 (4)	
H9	0.4930	0.4234	0.8360	0.021*	

### Atomic displacement parameters (Å^2^)

**Table:**

	*U*^11^	*U*^22^	*U*^33^	*U*^12^	*U*^13^	*U*^23^
W1	0.01293 (6)	0.01293 (6)	0.03153 (10)	0.000	0.000	0.000
Tb1	0.01091 (6)	0.01091 (6)	0.00970 (8)	0.000	0.000	0.000
O1	0.0165 (6)	0.0216 (7)	0.0134 (6)	0.0015 (5)	0.0022 (5)	0.0030 (5)
N1	0.0269 (10)	0.0294 (10)	0.0617 (14)	−0.0036 (8)	−0.0126 (9)	0.0106 (10)
C1	0.0180 (10)	0.0236 (10)	0.0435 (12)	−0.0035 (8)	−0.0054 (9)	0.0016 (10)
O2	0.0219 (7)	0.0334 (8)	0.0165 (6)	0.0156 (6)	−0.0023 (5)	0.0007 (6)
N2	0.0276 (10)	0.0317 (10)	0.0528 (12)	−0.0001 (8)	0.0092 (9)	−0.0084 (9)
C2	0.0233 (10)	0.0178 (9)	0.0422 (12)	−0.0016 (8)	0.0001 (9)	−0.0039 (9)
O3	0.0184 (7)	0.0235 (7)	0.0217 (7)	0.0064 (5)	−0.0021 (5)	0.0064 (5)
N3	0.0183 (8)	0.0235 (8)	0.0163 (8)	0.0101 (7)	−0.0004 (6)	−0.0014 (6)
C7	0.0223 (10)	0.0201 (10)	0.0322 (11)	0.0039 (8)	0.0008 (8)	0.0060 (8)
C6	0.0164 (9)	0.0200 (9)	0.0337 (11)	0.0009 (7)	−0.0031 (8)	0.0037 (8)
C5	0.0190 (9)	0.0165 (9)	0.0159 (8)	0.0048 (7)	0.0009 (7)	−0.0032 (7)
C4	0.0186 (9)	0.0199 (9)	0.0216 (9)	0.0009 (7)	−0.0002 (8)	0.0014 (8)
C3	0.0171 (9)	0.0268 (10)	0.0238 (10)	0.0021 (8)	−0.0004 (8)	−0.0016 (8)
C8	0.0170 (9)	0.0166 (9)	0.0137 (8)	0.0032 (7)	0.0022 (7)	−0.0039 (7)
C12	0.0218 (9)	0.0144 (9)	0.0220 (9)	−0.0014 (7)	−0.0017 (8)	0.0015 (7)
C11	0.0182 (9)	0.0201 (9)	0.0222 (9)	−0.0031 (7)	−0.0024 (8)	0.0019 (8)
N4	0.0167 (7)	0.0188 (8)	0.0133 (7)	0.0048 (6)	−0.0001 (6)	0.0008 (6)
C10	0.0202 (9)	0.0163 (9)	0.0169 (9)	0.0004 (7)	0.0037 (7)	0.0008 (7)
C9	0.0164 (9)	0.0180 (9)	0.0170 (9)	0.0005 (7)	0.0027 (7)	−0.0013 (7)

### Geometric parameters (Å, º)

**Table:**

W1—C2^i^	2.174 (2)	N3—C3	1.343 (3)
W1—C2^ii^	2.174 (2)	N3—C7	1.348 (3)
W1—C2^iii^	2.174 (2)	C7—C6	1.378 (3)
W1—C2	2.174 (2)	C7—H7	0.9300
W1—C1^i^	2.175 (2)	C6—C5	1.401 (3)
W1—C1^ii^	2.175 (2)	C6—H6	0.9300
W1—C1	2.175 (2)	C5—C4	1.396 (3)
W1—C1^iii^	2.175 (2)	C5—C8	1.484 (3)
Tb1—O2^iv^	2.3598 (13)	C4—C3	1.377 (3)
Tb1—O2^v^	2.3598 (13)	C4—H4	0.9300
Tb1—O2	2.3598 (13)	C3—H3	0.9300
Tb1—O2^vi^	2.3598 (13)	C8—C9	1.401 (3)
Tb1—O1^vi^	2.4104 (12)	C8—C12	1.402 (3)
Tb1—O1^iv^	2.4104 (12)	C12—C11	1.374 (3)
Tb1—O1	2.4104 (12)	C12—H12	0.9300
Tb1—O1^v^	2.4104 (12)	C11—N4	1.353 (2)
O1—H1B	0.8724	C11—H11	0.9300
O1—H1A	0.8755	N4—C10	1.352 (2)
N1—C1	1.157 (3)	C10—C9	1.379 (3)
O2—N3	1.336 (2)	C10—H10	0.9300
N2—C2	1.158 (3)	C9—H9	0.9300
O3—N4	1.3312 (19)		
			
C2^i^—W1—C2^ii^	74.13 (6)	O2^iv^—Tb1—O1^v^	72.77 (5)
C2^i^—W1—C2^iii^	116.94 (12)	O2^v^—Tb1—O1^v^	75.63 (5)
C2^ii^—W1—C2^iii^	74.13 (6)	O2—Tb1—O1^v^	146.09 (4)
C2^i^—W1—C2	74.13 (6)	O2^vi^—Tb1—O1^v^	73.41 (4)
C2^ii^—W1—C2	116.94 (12)	O1^vi^—Tb1—O1^v^	130.40 (4)
C2^iii^—W1—C2	74.13 (6)	O1^iv^—Tb1—O1^v^	72.76 (6)
C2^i^—W1—C1^i^	76.77 (8)	O1—Tb1—O1^v^	130.40 (4)
C2^ii^—W1—C1^i^	143.32 (7)	Tb1—O1—H1B	125.7
C2^iii^—W1—C1^i^	140.38 (7)	Tb1—O1—H1A	122.6
C2—W1—C1^i^	74.90 (8)	H1B—O1—H1A	101.0
C2^i^—W1—C1^ii^	74.90 (8)	N1—C1—W1	175.9 (2)
C2^ii^—W1—C1^ii^	76.77 (8)	N3—O2—Tb1	126.92 (10)
C2^iii^—W1—C1^ii^	143.32 (7)	N2—C2—W1	176.3 (2)
C2—W1—C1^ii^	140.38 (7)	O2—N3—C3	120.20 (16)
C1^i^—W1—C1^ii^	74.45 (6)	O2—N3—C7	119.38 (16)
C2^i^—W1—C1	143.32 (7)	C3—N3—C7	120.42 (16)
C2^ii^—W1—C1	140.38 (7)	N3—C7—C6	120.54 (18)
C2^iii^—W1—C1	74.90 (8)	N3—C7—H7	119.7
C2—W1—C1	76.77 (8)	C6—C7—H7	119.7
C1^i^—W1—C1	74.45 (6)	C7—C6—C5	120.81 (18)
C1^ii^—W1—C1	117.63 (12)	C7—C6—H6	119.6
C2^i^—W1—C1^iii^	140.38 (7)	C5—C6—H6	119.6
C2^ii^—W1—C1^iii^	74.90 (8)	C4—C5—C6	116.52 (17)
C2^iii^—W1—C1^iii^	76.77 (8)	C4—C5—C8	122.29 (17)
C2—W1—C1^iii^	143.32 (7)	C6—C5—C8	121.07 (17)
C1^i^—W1—C1^iii^	117.63 (12)	C3—C4—C5	120.87 (18)
C1^ii^—W1—C1^iii^	74.45 (6)	C3—C4—H4	119.6
C1—W1—C1^iii^	74.45 (6)	C5—C4—H4	119.6
O2^iv^—Tb1—O2^v^	140.48 (6)	N3—C3—C4	120.81 (18)
O2^iv^—Tb1—O2	96.56 (2)	N3—C3—H3	119.6
O2^v^—Tb1—O2	96.56 (2)	C4—C3—H3	119.6
O2^iv^—Tb1—O2^vi^	96.56 (2)	C9—C8—C12	116.87 (17)
O2^v^—Tb1—O2^vi^	96.56 (2)	C9—C8—C5	122.38 (17)
O2—Tb1—O2^vi^	140.48 (6)	C12—C8—C5	120.72 (17)
O2^iv^—Tb1—O1^vi^	73.41 (4)	C11—C12—C8	120.82 (17)
O2^v^—Tb1—O1^vi^	146.09 (4)	C11—C12—H12	119.6
O2—Tb1—O1^vi^	72.77 (5)	C8—C12—H12	119.6
O2^vi^—Tb1—O1^vi^	75.63 (5)	N4—C11—C12	120.38 (17)
O2^iv^—Tb1—O1^iv^	75.63 (5)	N4—C11—H11	119.8
O2^v^—Tb1—O1^iv^	72.77 (5)	C12—C11—H11	119.8
O2—Tb1—O1^iv^	73.41 (4)	O3—N4—C10	120.56 (15)
O2^vi^—Tb1—O1^iv^	146.09 (4)	O3—N4—C11	118.59 (15)
O1^vi^—Tb1—O1^iv^	130.40 (4)	C10—N4—C11	120.83 (16)
O2^iv^—Tb1—O1	146.09 (4)	N4—C10—C9	120.25 (17)
O2^v^—Tb1—O1	73.41 (4)	N4—C10—H10	119.9
O2—Tb1—O1	75.63 (5)	C9—C10—H10	119.9
O2^vi^—Tb1—O1	72.77 (5)	C10—C9—C8	120.71 (17)
O1^vi^—Tb1—O1	72.76 (6)	C10—C9—H9	119.6
O1^iv^—Tb1—O1	130.40 (4)	C8—C9—H9	119.6

Symmetry codes: (i) −*y*+3/2, *x*, *z*; (ii) −*x*+3/2, −*y*+3/2, *z*; (iii) *y*, −*x*+3/2, *z*; (iv) *y*−1/2, −*x*+1, −*z*+1; (v) −*y*+1, *x*+1/2, −*z*+1; (vi) −*x*+1/2, −*y*+3/2, *z*.

### Hydrogen-bond geometry (Å, º)

**Table:**

*D*—H···*A*	*D*—H	H···*A*	*D*···*A*	*D*—H···*A*
O1—H1*B*···O3^vii^	0.87	1.81	2.6695 (18)	167
O1—H1*A*···O3^viii^	0.88	1.89	2.7415 (18)	165

Symmetry codes: (vii) *y*, −*x*+3/2, *z*−1; (viii) *x*−1/2, *y*+1/2, −*z*+1.
